# Amino Acid Composition of Novel Plant Drinks from Oat, Lentil and Pea

**DOI:** 10.3390/foods9040429

**Published:** 2020-04-03

**Authors:** Astrid Bonke, Sander Sieuwerts, Iben Lykke Petersen

**Affiliations:** 1 Department of Food Science, University of Copenhagen, Rolighedsvej 26, 1958 Frederiksberg C, Denmark; astrid.bonke@food.ku.dk; 2 Brannatura ApS, Nygaardsvej 100, 8660 Skanderborg, Denmark; sander.sieuwerts@brannatura.com

**Keywords:** plant drink, capillary electrophoresis, *pisum sativum*, lens culinaris, avena sativa, oat, pea, lentil, protein, balanced amino acid profile, plant-based

## Abstract

Plant-based drinks (PBDs) as alternatives to milk is a fast-growing market in much of the western world, with the demand increasing every year. However, most PBDs from a single plant ingredient do not have an amino acid profile that matches human needs. Therefore, this study set out to combine plant ingredients to achieve a more balanced amino acid profile of novel plant drinks, by combining a high content of oat with the pulses pea (*Pisum sativum*) and lentil (*Lens culinaris*) in a solution. After removal of the sediment, the resulting plant drinks were composed of what could be kept in suspension. The amino acid and protein composition of the plant drinks were investigated with capillary electrophoresis, to identify the amino acids, and SDS-PAGE to assess the proteins present. The amino acid profile was compared against recommended daily intake (RDI). It was determined that the plant drinks with only oat and lentil did not have a strong amino acid profile, likely due to the higher pH of the lentil concentrate affecting which proteins could be kept in solution. Plant drinks with a combination of both lentil and pea, or only pea, added to the oat drink had an improved concentration of the amino acids that were otherwise in the low end compared to RDI. This includes a high content of phenylalanine, leucine and threonine, as well as a moderate amount of isoleucine, valine and methionine, and a contribution of histidine and lysine. An assessment of stability and sensory parameters was also conducted, concluding there was an advantage of combining oat with a legume, especially pea.

## 1. Introduction

Plant-based beverages, in particular milk alternatives, are a fast growing market in much of the western world, with the demand increasing every year [1]. Specifically, in Denmark, the sales volume of these plant-based drinks (PBDs) has increased by 30% in both 2015 and 2016 according to AC Nielsen marketing research, a trend that is predicted to continue [2], and a similar picture is seen across the western world [3]. This is due to a growing group of vegans, vegetarians, flexitarians and other people who choose more plant-based foods, as well as people specifically avoiding dairy for health or sustainability reasons [4,5].

Most of the PBDs on the market are of low nutritional value compared with bovine milk [6]. According to FAO (Food and Agricultural Organization of the United Nations), many countries recommend an intake of bovine milk of between one to three servings (corresponding to 200−600 mL milk), and one of the reasons for this recommendation is the content of high quality protein providing a balanced amino acid composition, meaning that it is close to what the average person needs [7]. So far, very few PBDs provide a suitable alternative to this. Most PBDs contain between 90%−98% water, and the product is very low in protein, often less than 1% of the total drink, with the exception of soy drink [8]. Additionally, the amino acid composition is less balanced, as most of the PBDs are from a single plant ingredient, and most plants are low in one or more of the essential amino acids. Therefore, it can be useful to combine plant ingredients in order to complement the individual amino acid profiles.

It is essential to have a good balance of amino acids in order to synthesize enough protein in the cells to keep the body healthy. Of the 20 amino acids needed to synthesize protein, nine are essential for adults and must be part of the dietary intake. If the dietary intake of amino acids is unbalanced, the amino acid that is most limiting becomes the bottleneck for the amount of protein synthesized.

Many grains are particularly low in the essential amino acid lysine. This includes oat, a popular grain used in PBDs, which contains only 575 mg lysine/100 g. With a recommended value of 2100 mg per day for an adult of 70 kg, it would require an intake of 365 g of oat, 1344 kcal, to cover the daily need for lysine with oat alone [9,10]. The most notable category of plant-based ingredient that can complement this lack of lysine is that of legumes. Legumes, such as lentils and peas, can have a lysine content approaching the daily recommended intake in just 100 g [10]. On the other hand, legumes tend to be low in the sulphur-containing amino acids cysteine and methionine, something that oat is more abundant in [10,11,12].

It may therefore be useful to use complementary plant ingredients in order to obtain a balanced essential amino acid profile, as well as to improve the stability and taste of the drink, as some PBDs based on only a single plant ingredient have been shown to be a challenge on these parameters [13] Hence, a combination of a grain and a legume could be an ideal and novel match for creating a good PBD with a balanced amino acid profile. This has led to the selection of whole grain oat, lentil protein concentrate and pea protein concentrate for this study. 

## 2. Materials and Methods 

### 2.1. Ingredients and Chemicals

Unless otherwise specified, all chemicals were purchased from Sigma-Aldrich. Betamalt, Optizym GA, Optizym A and Optizym BA enzymes were purchased from SternEnzym (Ahrensburg, Germany). SeeBlue^®^ Plus2 Prestained Standard from Invitrogen and Bolt™ LDS Sample Buffer and Bolt™ Sample Reducing Agent from Novex were purchased from Thermo Fisher Scientific (Waltham, MA, USA). Pure water obtained from a Milli-Q water purification system (Purification Pak, Millipore, Bedford, MA, USA) was used for all solutions and buffers.

### 2.2. Production of PBD Samples

To produce the PBDs, whole grain oat (oat flour, Belbake), lentil protein concentrate (51% protein, Biona) and raw pea protein concentrate (80% protein, Biona) were used. A total of 8 mixed PBDs were created for this experiment, based on oat supplemented with lentil protein concentrate and/or pea protein concentrate. The oat flour was ground and sieved through a 0.5 mw sieve, to ensure a smaller and more homogenous particle size. Additionally, three single-ingredient PBDs were made in three different versions, to test the effects of the treatments.

The eight PBDs based on mixed ingredients, as well as the nine single-ingredient PBDs, were made as shown in Table 1 and Table 2. This resulted in a total of 17 PBDs. 

Samples were heated to 65 °C for 45 min and then cooled to 55 °C before adding the enzymes. Optizym GA, Optizym A and Optizym BA were added at 0.0125 v%, whereas Betamalt was added at 0.02 w%. After adding the enzymes, samples were left to incubate for 3 h in a water bath with shaking at 300 rpm, whereafter enzymes were deactivated at 95 °C for 10 min and samples were cooled on ice for a minimum of 30 min. After cooling, samples were sieved through a 300-mic sieve to separate the liquid phase from the sediment, visibly containing mainly the bran particles of the whole grain oat. The single ingredient PBDs were used to assess the effect of this treatment. This was done by preparing them in three versions, one version that was simply mixed and sieved, another version that underwent the heat treatment as described, and one version that underwent the complete enzymatic treatment as prescribed above.

### 2.3. Amino Acid Composition

The samples were freeze dried at 0.1 mbar and −30 °C for 3 h with a Buch Holm CHRIST Gamma 1−16 LSCplus freeze dryer with a Buch Holm CHRIST LyoCube (Herlev, Denmark). The dried samples were hydrolyzed under 65 millitorr vacuum for 2 min and with 200 μl 6 M HCL/1% phenol three times and left at 105 °C for 24 h in a Waters Picotag (Milford, MA, USA) workstation.

From the hydrolyzed samples, 50 µL was reconstituted twice in 75 µL abs. alcohol and 75 µL 100 mM sodium borate (Na_2_B_4_O_7_) to a 400 µL solution. Following, 50 µL was mixed with 25 µL internal standard (norvaline, 4.6 µmol/mL). After hydrolysis, samples were derivatised using 75 µL 70 mM Sangers reagent (1-fluoro-2,4-dinitrobenzene). The samples were then heated, dried and reconstituted in 200 µL 20% MeOH.

The High Performance Capillary Electrophoresis (HPCE) instrument used was the 3D CE DE01602655 from Hewlett Packard (now HP^®^) (Palo Alto, CA, USA), and the 75-µm capillary tube, cut to 80 cm, was from Agilent Technologies (Santa Clara, CA, USA). The method was set for samples derivatized with DNFB, at 50 °C, −18 kV, UV detection at 360 nm, and 75 min running time. The injection time was set to 2 s at 50 mbar. 

### 2.4. Other Analyses

For a qualitative protein assessment, SDS-PAGE (Sodium Dodecyl Sulfate Polyacrylamide Gel Electrophoresis) BOLT™ 4%–17% Bis-Tris-Plus with 15 1.0 mm wells from Invitrogen by Thermo Fisher Scientific (Waltham, MA, USA) was used. Samples from the sediment and supernatant of each of the PBDs were analyzed on three different gels. In order to standardize, the same amount of protein was loaded in each well.

For the determination of nitrogen, and thus the protein content, it was first necessary to know the dry matter content, measured with the HB43 Halogen Moisture Analyzer from Mettler Toledo (Columbus, OH, USA). 

The samples were packed in 37 × 37 mm tinfoil squares from Elemental Microanalysis (Oakhampton, UK), in triplicates of approximately 25.00 mg, before being transferred for analysis on the CHNS Elemental Analyzer, Vario MACRO Cube from Elementar (Langelsebold, Germany). A sulfanilamide reference was obtained from Elemental Microanalysis (Oakhampton, UK), and loaded to the CHNS as well. The nitrogen content measured here was multiplied by 6.25 [14] to obtain the protein content.

For the pea samples, the content of amino acids measured by the HPCE (High-Performance Capillary Electrophoresis) was used to calculate the total protein content.

For pH measurement, the 827 pH lab pH-meter from Metrohm (Herisau, Switzerland) was used. 

The dry matter and total nitrogen in the PBDs after removal of the sediment by sieving were used in the calculation to determine the amino acid content. This method was used for all except the pea samples, where the total of the amino acids was used to estimate protein content. 

The stability of the sieved PBDs was assessed by observing 5 mL of the PBDs in identical test tubes in a fridge (5 °C) for 7.5 h, and documented every 30 min, to asses sedimentation and stability of emulsion.

Additionally, a small sensory assessment was made on three of the PBDs, by inviting six people to taste the PBDs which had been given 3-digit codes and placed randomly in front of the participants. The participants were all familiar with PBDs, to counteract neophobia. The participants then made notes on various sensory aspects, and a small discussion summarized the findings.

## 3. Results

### 3.1. The Amino Acids

The content of each amino acid in 500mL of the PBD can be compared to the reference daily intake (RDI) from the WHO/FAO/UNU for a 70 kg adult [9], as depicted in Table 3 and Table 4.

The raw samples did not undergo heating or enzymatic treatment. The heated samples only underwent the heat treatment, and the enzyme samples underwent both heat treatment and enzymatic treatment. The protein percentage is the protein content found in the supernatant after removal of sediment. Protein content is calculated from the N content, using the conversion factor of 6.25, except for the pea samples, where the protein content was calculated from the total mass of the amino acids in the sample

### 3.2. Mass Balance and pH

The mass balance was determined as the fraction of the initial mass, total dry matter, protein and moisture ending up in the supernatant and in the sediment after the final sieving. This is presented in Table 5. Further information about the pH of each sample is also included in the same table.

### 3.3. SDS-PAGE

**Figure 1:**
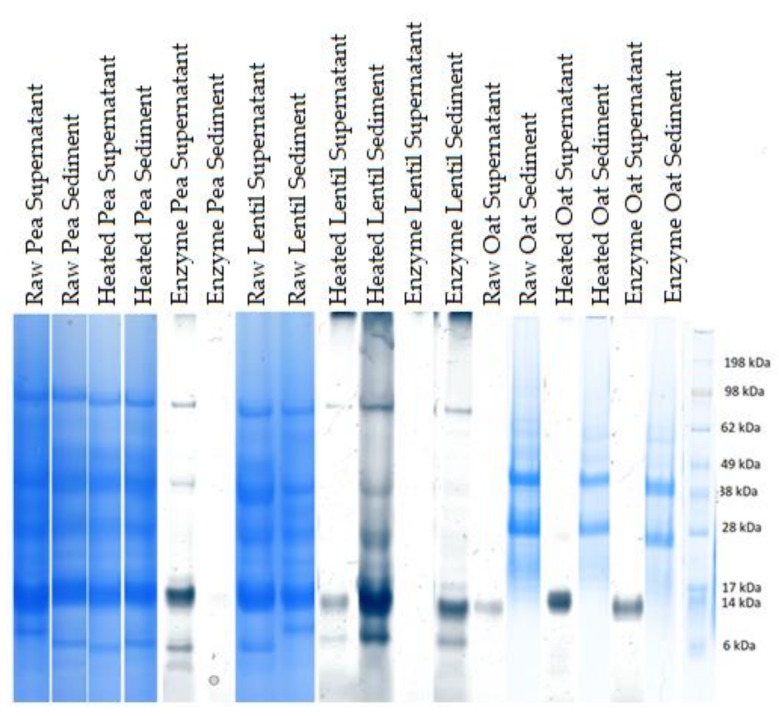
Major protein sizes as determined by SDS-PAGE for the single-ingredient samples, analyzed under reducing condition. Furthest left lane: MW ladder.

**Figure 2:**
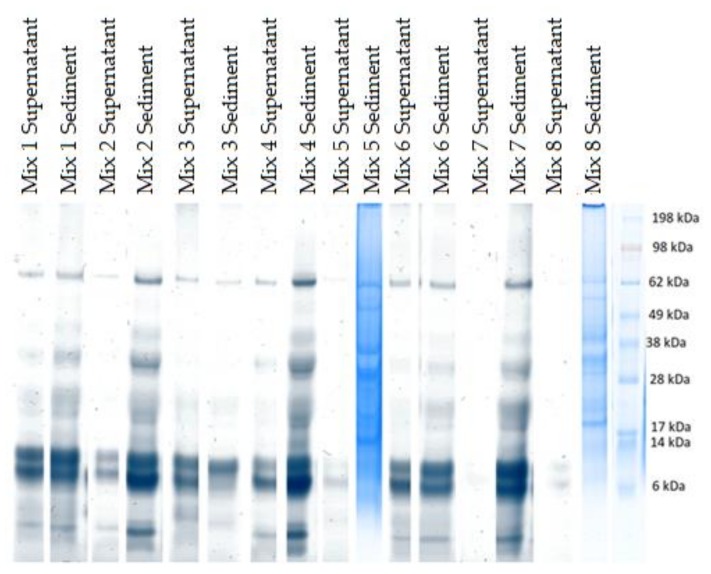
Major protein sizes as determined by SDS-PAGE for the mixed samples. Analyzed under reducing condition. Furthest left lane: MW ladder.

The sizes and approximate relative abundances of major proteins in the samples were determined by SDS-PAGE, as depicted in Figure 1 and Figure 2. 

### 3.4. Stability Assessment 

Notable observations of the stability assessment are presented here. Some sedimentation is observed already after t = 0.5 h, on all three of the single ingredient samples. In the pea samples, a sediment is clearly observed at t = 1.5 h. The rest of the PBD, is, however, still milky in appearance. For the oat sample, it can be seen that this appears to have created a gel that takes up almost the entire space of the PBD, with a clearly separated layer on top. The same effect can be seen to a much lesser extent in Mix 5, Mix 7 and Mix 8, each having a clear layer of liquid on top, and the remainder of the drink appearing unchanged.

For all the PBDs, syneresis and sedimentation was observed within the first 2.5 h, and there is not much more separation after the assessment has run to its end.

### 3.5. Sensory Assessment

The sensory assessment led to descriptions of the PBDs regarding appearance, smell, flavor and mouthfeel. For Mix 3, small particles were visible in the PBD, and it appeared thick and yellowish, with a sweet and mild smell. The flavor was reported as sweet, chocolaty and dessert-like. The mouthfeel was thick and slightly sticky. Mix 4 appeared milky in color and again with visible particles. The smell was mild and grainy. The flavor was reported as being strong, and with distinct flavors of beans and flour. The mouthfeel was grainy and sticky. The last assessed PBD was Mix 6, which was also reported as milky in color and had an appearance of thin viscosity, and with small particles visible. The smell was mildly cereal-like, and the tasted strongly of cereal. The mouthfeel was reported as dry, grainy and felt like it had a thin viscosity. 

## 4. Discussion

A major observation is the large difference seen between the amounts of protein in the liquid phase from raw, heated and enzyme-treated single-ingredient samples. For lentil and oat, it was found that the raw samples had the lowest amount of the protein retained in the supernatant. For the heat-treated and heat + enzyme-treated samples, almost equal amounts of protein were retained in the supernatant, indicating that their proteins dissolve better at higher temperatures and that saccharification of starch does not have much influence on protein solubility. For pea, on the other hand, there was low protein content in the heated sample, even though pea and lentil in many respects are very similar. The fact that the pea protein ingredient had been concentrated to have so little carbohydrate left could be one of the reasons for the observed difference in how the samples react to heat. For the more starch-rich ingredients, oat and lentil, the heating makes the starches gelate thereby making the sample very viscous. A very viscous sample can keep insoluble components, such as proteins, in suspension [3,13], whereas for the pea, there is very little starch to gelate and make the sample viscous, giving sedimentation of low-thermostable proteins during the heating steps in the process [15,16]. This is also confirmed when looking at the results for the stability assessment. The pea samples appear to quickly form a large sediment, whereas the other two PBDs keep the emulsion. Due to the alkalinity of the pea PBD compared to the two others, this sediment is not likely to be due to protein precipitation, but rather the carbohydrates not being stable, as most of them are insoluble fibers [11]. For the oat, the gel is likely to be the amylopectin and amylose that has created a network with pockets of the other insoluble components, leaving the remainder of the drink clear [13]. This indicates that the starch’s gelling abilities are not eliminated with the enzyme treatment, as others have thought would be the case [17]. For the enzyme-treated samples, all three single-ingredient drinks have a large part of their protein in the drink, above 90% for lentil and pea, rather than in the sediment. This is likely due to the reduction of the overall amount of insoluble parts, as the enzymes have broken down some of the insoluble starches. This then allows more of the otherwise insoluble components to be kept in the suspension, as there is no longer an oversaturation by starch. This is consistent with previous studies showing the effect of oversaturation [8,15]. This is also seen by the stability assessment, where there is much less sedimentation for these PBDs than the untreated ones. 

When comparing the drinks with the RDI set forth by WHO (World Health Organization)/FAO [9], the drinks that perform well are Mix 1, Mix 3, and Mix 7, having at least a third of the RDI for each amino acid in 500mL, except for amino acids that may not be measured due to the method. This includes amino acids that are destroyed or converted during acid hydrolysis, such as cysteine and tryptophan as well as glutamine and asparagine [18]. Additionally, the long duration of the acid hydrolysis may have also made some amino acids, such as isoleucine, valine and lysine, react with the starch, and made them appear in smaller quantities [19]. Furthermore, there were signs of co-elution of valine and methionine, such as is seen with various other amino acids in previous studies [18,19,20,21]. There are also amino acids with peaks close to the limit of detection, and they may be underestimated, such as histidine and some of the derivates of lysine [22].

Mix 1 and Mix 3 are mixes with a high content of oat, and therefore a high starch content. It is therefore likely that the high viscosity that the higher concentration of starch in these samples creates was able to keep otherwise non-soluble proteins in suspension, thereby creating the more well-balanced amino acid composition. This can also be seen in both the stability assessment and the mass balance, where a larger portion of starches and proteins are kept in what appears to be very viscous, emulsified PBD.

Discussion of which proteins are kept in solution and suspension requires knowledge about which proteins are found in the ingredients. Previous studies have provided this information, as seen in Table 6.

The proteins that are likely to be insoluble in PBDs, given the pH of the solutions, are avenin, albumin 1 and legume globulin [23,25,29]. The latter two are high in isoleucine [23,29], and they are all high in phenylalanine [23,26,29], amino acids that are found less in the suspensions of the mixes with less starch compared to, in particular, mixes 1, 2 and 3. Avenin is also especially high in glutamine [29], which can then be compared to the amino acid profile, where we see that these three mixes also have a very high content of glutamine/glutamic acid, i.e., above 600 mg/100 g. This confirms that they are likely to have a high content of avenin. Reviewing the SDS-PAGE results in Figure 1 also confirms a presence of avenin (33 kDa) in these mixes. For Mix 3 especially, it was found that avenin is present more in the drink than the sediment, confirming the amino acid measurement observations. Mixes 6 and 7 also have balanced amino acid profiles. They are, however, on the low side for tyrosine, an amino acid found especially in albumin 2 as well as oat globulin [24,30]. The fact that these mixes are low in oat content explains that these mixes have a low content of this protein. This is confirmed when reviewing the SDS-PAGE, where the bands appear weaker than those for mixes 1, 2 and 3. Considering phenylalanine, this amino acid is present in very high concentrations in some of the PBDs, namely the raw pea drink, the heated lentil drink, the enzyme + heat treated lentil drink, as well as Mix 6 and Mix 7, having respectively equal amounts of lentil and pea, and more lentil than pea. Albumin 2 is the protein with the highest phenylalanine content, at 8.2% of the amino acids in the legumes, and it is therefore this protein that is likely to be present at higher quantities in the PBDs with high phenylalanine content [24]. To explain why some of the PBDs have more of this protein, it is relevant to both consider the contents of the protein in the ingredients and their solubility at different pHs, which can determine whether the protein stays in the solution, or if it is precipitated and removed with the sediment. Albumin 2 is the protein with the lowest isoelectric point, at 5.16 [24]. It is therefore unlikely to stay in the solution of PBDs with a low pH. The PBDs with the lowest pH are the ones with the most lentil protein added, although still above the isoelectric point. Previous studies have also found low solubility at pHs higher than the pI for the protein, and have suggested this to be due to the formation of insoluble protein complexes when the protein powder is prepared with an isoelectric protein precipitation method [31]. It is seen that the samples containing very little lentil do not contain much phenylalanine either, even though the pH is higher, and should therefore be more ideal with respect to keeping albumin 2 in solution. Therefore, it could be an explanation that the lentil protein is high in albumin 2, but that it has low solubility in mildly acidic circumstances, a condition relieved by the more alkaline pea protein ingredient. The molecular weight of albumin 2 is around 25 kDa [24], and at this size, the SDS-PAGE shows a band for all the samples, both supernatant and sediment, suggesting that the ingredients are indeed rich in this protein. The SDS-PAGE results do not allow for detailed differentiation, but comparing the drink/suspension with the highest phenylalanine content, Mix 6, with that with the lowest phenylalanine content, Mix 5, a clearer band at 25 kDa is observed for Mix 6 than Mix 5. Comparing this to the mass balances, we see that the two samples are otherwise comparable, and the difference in their SDS-PAGE and amino acid compositions is therefore indeed likely to be due to the combinations in types of proteins than quantities of proteins distributed in the supernatant and sediment. However, this hypothesis is challenged when looking at another amino acid that is present at high concentration in albumin 2: tyrosine, which is reported to be present at 6.9% [24]. The tyrosine content differs vastly between the different mixes, and contrary to phenylalanine, it has a low standard deviation in all the triplicate samples. However, it is too low in concentration to be detected in Mix 6 and 7, the two mixes where the high phenylalanine content was used to draw a tentative conclusion that these must be high in albumin 2. This suggests that the phenylalanine content may also come from another protein: the avenins from oat [29]. Avenin also has 8.20% phenylalanine, and while having a significantly higher isoelectric point of 6.69, it is still less precipitated in the samples with higher pH, such as Mix 6 and 7. The composition of avenin differs significantly in tyrosine content from albumin 2, having only 1.4% [24]. Nevertheless, the overall amino acid contribution of the oat, and therefore avenin, is almost a factor of three smaller than that of the legumes [29]. Looking at the SDS-PAGE results, however, we see as a significant band at 32 kDa for avenin, as was observed for albumin 2. It is therefore likely that the high phenylalanine content is due to a combination of high avenin and albumin 2 content, rather than one of the proteins alone. Finally, it can be concluded that a serving size of 500 mL of Mix 1, Mix 3 or Mix 7 would, according to the RDI, provide an average person (70 kg) with a third or more of the isoleucine, valine and methionine they need for the day, around 50% or more of their need for phenylalanine, leucine and threonine, and a smaller amount (≤ 10%) of histidine and lysine, the latter which has been found to be underestimated by the amino acid analysis.

The sensory assessment showed positively charged descriptions of Mix 3, one the PBDs with a high content of oat. This is consistent with other findings, which shows that oat PBD is a positively evaluated PBD [3]. The fact that the nutritionally beneficial addition of a legume did not deter this evaluation is a positive finding in this study. However, for all the PBDs used in the sensory evaluation, some off flavors were found, which is typical for PBDs such as these, containing oat and legumes [3,13]. Looking at the samples individually showed some notable differences. It was found that the lentil samples had a stronger bean flavor. This is likely due to the extra lentil carbohydrate, meaning that more lentil powder was added to get the same protein amount, and therefore more lentil flavor was added. This off flavor was not present in the samples with both pea and lentil, or only pea added to the oat drink, indicating that the more concentrated pea did not add detectable amounts of bean flavor.

For the appearance, most of the samples were notable for having a grainy appearance and visible particles. This is likely due to the particle size, and the enzymes not having been able to fully access the larger starches, which has previously shown to be of great importance to the overall effectiveness of the enzyme treatment [8]. This is also observed for the mouthfeel of many of the samples, especially for Mix 6, which was also noted to have a dry mouthfeel, which is a common problem with PBDs made from low fat ingredients [3,8]. Mix 6 is the sample with the least percentage of oat. This indicates that the oat had a positive effect on the smoothness of the mouthfeel in the samples, and that the lentil protein on its own had an unpleasant mouthfeel. The positive mouthfeel of enzymatic treated oat, compared to other plant sources, has been shown before, so this is consistent with the literature [3,8]. For the color perception, it was found that the most white and milky appearance was in the samples with the no pea protein present. This indicates that the pea protein is the component providing the most altered color. This is confirmed by observations in the lab, showing a strong yellow color when the pea protein powder was wetted. For the appearance of the samples, it was found that Mix 3 is the one that appear to be of highest viscosity. Part of the purpose of using enzymes was to be able to increase the dry matter content without notably increasing the viscosity. However, the participants did not state the thickness of the sample as a negative trait. The apparent higher viscosity of Mix 3 is confirmed by this sample having the highest dry matter content. This is not because more of the ingredients were added to the sample, but more were retained in the suspension when the sediment was removed. This is likely because more of the starch consists of amylopectin than in the samples where some of the starch is from the more amylose-rich lentil [16,32]. The amylopectin was likely able to create a strong gel network that kept more of the dry matter in the plant milk, as well as increased the viscosity of the final sample.

## 5. Conclusions

It was found that the most ideal mixes for a complete amino acid composition were Mix 1 (1.1% oat protein in the raw ingredients, 1.5% protein from pea and lentil each), Mix 3 (1.1% oat protein, 2.9% pea protein), and Mix 7 (0.8% oat protein, 1.1% pea protein, 2.1% lentil protein), contributing significantly with essential amino acids compared to RDI recommendations. The results furthermore indicate that the presence of starch in the samples, as well as a neutral to slightly alkaline pH, improves recovery of proteins in suspension when removing insoluble particles above 300 microns. Furthermore, the viscosity created by the starches, and the emulsion that follows it, has a great effect on both protein suspendability as well as overall dry matter kept in the PBD. Sensorially, the mixed PBDs with a high content of oat were evaluated positively, showing another benefit of combining the legume with another, more sensorially pleasing, ingredient.

## Figures and Tables

**Table 1:** Added ingredients and their protein contributions to the mixed PBDs.

Percent Added	Mix	Mix	Mix	Mix	Mix	Mix	Mix	Mix
Ingredient w/w	1	2	3	4	5	6	7	8
Lentil concentrate (51%)	2.9	5.7	0.0	6.3	0.0	3.1	4.2	2.2
Pea isolate (80%)	1.8	0.0	3.7	0.0	4.0	2.0	1.3	2.6
Whole grain oat flour	8.0	8.0	8.0	6.0	6.0	6.0	6.0	6.0
Total content of ingredients	12.7	13.7	11.7	12.3	10.0	11.1	11.5	10.8
Protein contribution from the ingredients (% w/w)								
Lentil concentrate (51%)	1.5	2.9	0.0	3.2	0.0	1.6	2.1	1.1
Pea isolate (80%)	1.5	0.0	2.9	0.0	3.2	1.6	1.1	2.1
Whole grain oat flour	1.1	1.1	1.1	0.8	0.8	0.8	0.8	0.8
Total protein concentration	4	4	4	4	4	4	4	4

**Table 2:** Added ingredients and their protein contribution to the single ingredient PBDs.

Percent Added	Oat	Lentil	Pea	Oat	Lentil	Pea	Oat	Lentil	Pea
Ingredient w/w	Raw	Raw	Raw	Heated	Heated	Heated	Enzyme	Enzyme	Enzyme
Lentil concentrate (51%)	0.0	7.8	0.0	0.0	7.8	0.0	0.0	7.8	0.0
Pea isolate (80%)	0.0	0.0	5.0	0.0	0.0	5.0	0.0	0.0	5.0
Whole grain oat flour	10.0	0.0	0.0	10.0	0.0	0.0	10.0	0.0	0.0
Total content of ingredients	10.0	7.8	5.0	10.0	7.8	5.0	10.0	7.8	5.0
Protein contribution from the ingredients (% w/w in the PBD formulation)									
Lentil concentrate (51%)	0.0	4.0	0.0	0.0	4.0	0.0	0.0	4.0	0.0
Pea isolate (80%)	0.0	0.0	4.0	0.0	0.0	4.0	0.0	0.0	4.0
Whole Grain Oat Flour	1.3	0.0	0.0	1.3	0.0	0.0	1.3	0.0	0.0
Total protein content	1.3	4.0	4.0	1.3	4.0	4.0	1.3	4.0	4.0

**Table 3:** The essential amino acids measured in the supernatant of the single ingredient PBDs.

	Raw Oat		Raw Lentil		Raw Pea	
	0.6% protein		1.6% protein		2.23% protein	
	mg/100 mL	% of RDI in 500 mL	mg/100 mL	% of RDI in 500 mL	mg/100 mL	% of RDI in 500 mL
Isoleucine	0	0	46	16	87	31
Leucine	0	0	91	17	152	28
Lysine	0	0	34	8	51	12
Valine/Methionine	35	6	67	12	116	20
Phenylalanine	0	0	214	77	308	118
Tyrosine	0		55		104	
Threonine	0	0	93	44	86	41
Histidine	0	0	333	24	14	10
	**Heated Oat**		**Heated Lentil**		**Heated Pea**	
	0.5% protein		3.7% protein		0.96% protein	
	mg/100 mL	% of RDI in 500 mL	mg/100 mL	% of RDI in 500 mL	mg/100mL	% of RDI in 500 mL
Isoleucine	17	6	0	0	49	18
Leucine	39	7	124	23	86	16
Lysine	63	15	130	31	29	7
Valine/Methionine	17	3	151	26	66	11
Phenylalanine	63	21	576	185	175	66
Tyrosine	12		70		57	
Threonine	33	16	1073	511	48	23
Histidine	12	9	0	0	8	6
	**Enzyme Oat**		**Enzyme Lentil**		**Enzyme Pea**	
	0.5% protein		3.6% protein		2.64% protein	
	mg/100 mL	% of RDI in 500 mL	mg/100 mL	% of RDI in 500 mL	mg/100 mL	% of RDI in 500 mL
Isoleucine	4	2	122	44	92	33
Leucine	23	4	257	47	160	29
Lysine	0	0	137	33	53	13
Valine/Methionine	23	4	171	30	122	21
Phenylalanine	101	30	385	161	323	123
Tyrosine	4		178		106	
Threonine	113	54	146	70	89	42
Histidine	0	0	92	66	15	11

mg/100 mL represents the amount of the amino acid found in the finished and sieved drink. % of RDI in 500 mL represents the amount of the recommended daily intake [9] of a 70 kg adult that would be covered with an intake of 500 mL of the plant drink. Note that methionine and cysteine have a pooled RDI, and the reference here therefore includes cysteine, despite this amino acid not being measurable by this method.

**Table 4:** The essential amino acids measured in the mixed samples.

	Mix 1 3.7% Protein		Mix 2 3.4% Protein		Mix 3 4.1% Protein		Mix 4 3.1% Protein	
	mg/100 mL	% of RDI in 500 mL	mg/100 mL	% of RDI in 500 mL	mg/100 mL	% of RDI in 500 mL	mg/100 mL	% of RDI in 500 mL
Isoleucine	109	39	115	41	142	51	70	25
Leucine	253	46	267	49	380	70	175	32
Lysine	82	20	75	18	135	32	80	19
Valine/Methionine	217	38	146	25	290	51	42	7
Phenylalanine	194	75	84	25	120	45	92	63
Tyrosine	67		4		38		130	
Threonine	140	67	86	41	95	45	161	77
Histidine	17	12	78	56	14	10	39	28
	**Mix 5 3.8% protein**		**Mix 6 3.8% protein**		**Mix 7 4.3% protein**		**Mix 8 3.9% protein**	
	**mg/100 mL**	**% of RDI in 500 mL**	**mg/100 mL**	**% of RDI in 500 mL**	**mg/100 mL**	**% of RDI in 500 mL**	**mg/100 mL**	**% of RDI in 500 mL**
Isoleucine	118	42	93	33	104	37	105	37
Leucine	223	41	259	47	284	52	265	48
Lysine	88	21	41	10	92	22	88	21
Valine/Methionine	171	30	406	71	211	37	191	33
Phenylalanine	71	49	585	167	549	157	76	42
Tyrosine	101		0		0		72	
Threonine	178	85	280	133	305	145	169	80
Histidine	32	23	0	0	0	0	0	0

Protein content is calculated from the N content, using the conversion factor of 6.25. mg/100 mL represents the amount of the amino acid found in the finished and sieved drink. % of RDI in 500 mL represents the amount of the recommended daily intake [9] of a 70 kg adult that would be covered with an intake of 500 mL of the plant drink. Note that methionine and cysteine have a pooled RDI, and the reference here therefore includes cysteine, despite this amino acid not being measurable by this method.

**Table 5:** Fraction of sample mass, fraction of dry matter (DM), fraction of protein and fraction of moisture kept in suspension. Additionally, the pH of each of the finished drinks/suspensions is listed.

-	Raw Pea	Raw Lentil	Raw Oat	Heated Pea	Heated Lentil	Heated Oat	Enzyme Pea	Enzyme Lentil	Enzyme Oat	Mix 1	Mix 2	Mix 3	Mix 4	Mix 5	Mix 6	Mix 7	Mix 8
Fraction of total content that is kept in suspension in the supernatant																	
Mass	65%	85%	65%	52%	64%	45%	63%	67%	59%	59%	62%	60%	61%	56%	61%	58%	66%
DM	27%	24%	19%	18%	26%	19%	28%	26%	22%	28%	25%	28%	25%	27%	27%	30%	28%
Protein	95%	0%	46%	8%	93%	38%	98%	90%	38%	93%	85%	100%	78%	95%	95%	100%	98%
Moisture	58%	91%	81%	52%	67%	56%	55%	71%	71%	78%	92%	73%	82%	63%	74%	69%	77%
pH	6.49	7.66	6.32	6.47	7.55	6.30	6.20	7.34	6.24	6.47	6.28	6.98	6.21	7.01	6.53	6.42	6.66

Note that it is not the percentage of the water, protein and dry matter found in the samples, but a percentage of how much of the original mix of water and ingredients that remains in the drink/supernatant.

**Table 6:** Proteins in Pea, Lentil and Oat, with size, weight and pI.

-	Pea and Lentil Proteins						Oat Proteins	
-	**Albumin 1^1^**	**Albumin 2^2^**	**Globulin^3^**	**Legumin^4^**	**Vicilin^5^**	**Lectin^6^**	**Avenin^7^**	**Globulin^8^**
Num. of amino acids	130	231	488	520	459	275	281	551
Molecular weight	14 kDa	25 kDa	53 kDa	59 kDa	52 kDa	30 kDa	33 kDa	62 kDa
Theoretical pI	6.68	5.16	6.84	6.21	5.39	4.90	6.69	9.20

^1^[23]; ^2^[24]; ^3^[25]; ^4^[26]; ^5^[27]; ^6^[28]; ^7^[29]; ^8^[30]^.^ Data based on selected references, as indicated.
